# Light Emitting Diodes based Photoacoustic Imaging and Potential Clinical Applications

**DOI:** 10.1038/s41598-018-28131-4

**Published:** 2018-06-29

**Authors:** Yunhao Zhu, Guan Xu, Jie Yuan, Janggun Jo, Girish Gandikota, Hakan Demirci, Toshitaka Agano, Naoto Sato, Yusuke Shigeta, Xueding Wang

**Affiliations:** 1 Department of Biomedical Engineering, University of Michigan, Ann Arbor, Michigan 48109 USA; 2 Department of Electronic Science and Engineering, Nanjing University, Nanjing, Jiangsu 21000 China; 3 Department of Radiology, University of Michigan, Ann Arbor, Michigan 48109 USA; 4 Kellogg Eye Center, University of Michigan, Ann Arbor, Michigan 48109 USA; 5 PreXion Corporation, Tokyo, 1010041 Japan

## Abstract

Using low cost and small size light emitting diodes (LED) as the alternative illumination source for photoacoustic (PA) imaging has many advantages, and can largely benefit the clinical translation of the emerging PA imaging technology. Here, we present our development of LED-based PA imaging integrated with B-mode ultrasound. To overcome the challenge of achieving sufficient signal-to-noise ratio by the LED light that is orders of magnitude weaker than lasers, extensive signal averaging over hundreds of pulses is performed. Facilitated by the fast response of the LED and the high-speed driving as well as the high pulse repetition rate up to 16 kHz, B-mode PA images superimposed on gray-scale ultrasound of a biological sample can be achieved in real-time with frame rate up to 500 Hz. The LED-based PA imaging could be a promising tool for several clinical applications, such as assessment of peripheral microvascular function and dynamic changes, diagnosis of inflammatory arthritis, and detection of head and neck cancer.

## Introduction

As an emerging biomedical imaging technology, photoacoustic (PA) imaging, also named as optoacoustic imaging, has been rapidly developed in the past decades and extensively explored for potential applications in preclinical and clinical settings^1–5^. By illuminating a biological sample with nanosecond pulsed light and then detecting the light-generated ultrasound (US) signal as a result of thermoelastic expansion, a 2D or 3D map reflecting the optical-absorption contrast in the sample can be produced *via* different image reconstruction methods. By combining the merits of both light and ultrasound, PA imaging is capable of presenting highly-sensitive optical spectroscopic contrast in subsurface biological tissue at high ultrasonic spatial resolution. The spatial resolution of PA imaging is not only determined by the ultrasonic detection bandwidth of the system but also limited by the pulse duration of the illumination light source. To achieve higher spatial resolution, shorter light pulse is desired^6^. In addition, to satisfy the stress confinement which is crucial for producing PA signals efficiently, the duration of the light pulse needs to be less than the acoustic transit time across the scale of spatial resolution^6^. For a PA imaging system working with a 10-MHz center frequency transducer array with a targeted spatial resolution of 150 μm, the stress confinement requires a light source enabling pulse duration of less than 100 nanoseconds. When the targeted spatial resolution is higher, the duration of the light pulse needs to be further reduced. In tomographic PA imaging where the target biological sample is usually illuminated entirely by a broad light beam, sufficient light energy of a few to hundreds of millijoules per pulse needs to be delivered to the sample within a pulse duration from a few to several tens of nanoseconds. To satisfy this high demand, almost all of the current PA imaging systems are based on Ti:Sapphire lasers, optical parametric oscillators (OPO), or dye lasers pumped by powerful Q-switched Nd:YAG lasers which are defined as class IV. Although these lasers work fine for PA systems developed or used in laboratories, their high cost, large footprint, and less mobility could substantially hinder the translation of this promising imaging technology from bench to clinic.

In recent years, laser diode (LD) based PA imaging systems have been employed in several *in vivo* preclinical and clinical studies^7–11^. For example, it has been reported that a LD light source (Quantel, Bozeman, MT) can provide 1.4 mJ pulse energy at up to 7 kHz pulse repetition frequency^8,10^. Same as class-IV lasers, the emission from LD is also coherent. This potential focusing of the light beam at the retina raises concern of eye safety when implementing PA imaging using LD or class-IV laser. To protect eye, anyone within the operation area has to wear laser safety glasses, which may affect the willingness of patients and physicians to adopt the new PA imaging technology.

The recent advancement of light emitting diode (LED) technology offers a unique opportunity to solve the challenges in clinical applications of PA imaging^12,13^. Compared to expensive class-IV laser systems, the LED-based light source is much lower in both owning and operating costs. The lower costs together with the significantly reduced footprints of the LED light source can make PA imaging a practical option for point-of-care screening or diagnosis of a variety of diseases. Furthermore, unlike class-IV laser systems which need to be placed in dedicated spaces securing safety and operation requirements (*e.g*., light shielding, high electric power, and air or water cooling), LED light source can operate in almost any place, including resource deficient settings such as on battlefields or in ambulances. At the light fluence we are working with, there is no need for wearing laser safety glasses for anyone within the operation area.

The biggest challenge for LED-based PA imaging is to produce sufficient signal-to-noise ratio (SNR) in biological samples. During limited pulse duration of 100 ns or less, LED, even working as a group such as a 2D panel, can only deliver light energy that is about two orders of magnitude lower than that from a class-IV laser. Without performing signal averaging, this low pulse energy will not be able to produce detectable PA signal from biological samples even at the surfaces. To overcome this, in a previous study using a single LED element delivering 10 µJ per pulse with pulse repetition rate of 500 Hz, Allen *et al*. had to perform over 5,000 times of signal averaging when tried to image tubes filled with human blood^12^. Briefly reported before^14^, we have developed 2D LED array panels that each can deliver up to 200 µJ of pulsed light at 850-nm wavelength. Another research group examined the characteristics of our system, such as the LED beam profile, and the spatial resolution and the imaging depth. That study also investigated the performance of our system in resolving exogenous contrast agents^13^.

Very short pulse duration of tens of nanoseconds was made possible by the fast response of the LED and the high-speed driving. Taking advantage from the high pulse repetition rate up to 16 kHz, extensive signal averaging from dozens to hundreds of pulses can be performed. In this way, the LED-based PA imaging system is able to achieve SNR comparable to those in laser-based PA imaging systems without sacrificing the capability of performing real-time imaging^8,15^. As demonstrated in this work, the LED-based PA imaging integrated with a conventional B-scan ultrasound is capable of dual-modality imaging of biological samples at a frame rate up to 500 Hz, which is sufficient to present and evaluate the pulsations of arteries and the blood reperfusion in subsurface tissue with a depth over 5 mm. After evaluating the specifications of our LED light sources and examining the performance of our LED-based PA imaging system, we have explored the potential of this system for clinical applications, including assessment of peripheral microvascular function and dynamic changes, and diagnosis of inflammatory arthritis, and the detection of head and neck cancer. All these pathological conditions are associated with structural and functional changes in microvessels, and could benefit from the low-cost, small footprint, safe, and patient-friendly PA imaging system equipped by the LED light source.

## Results

### Imaging of peripheral microvasculature and function

**Figure 1:**
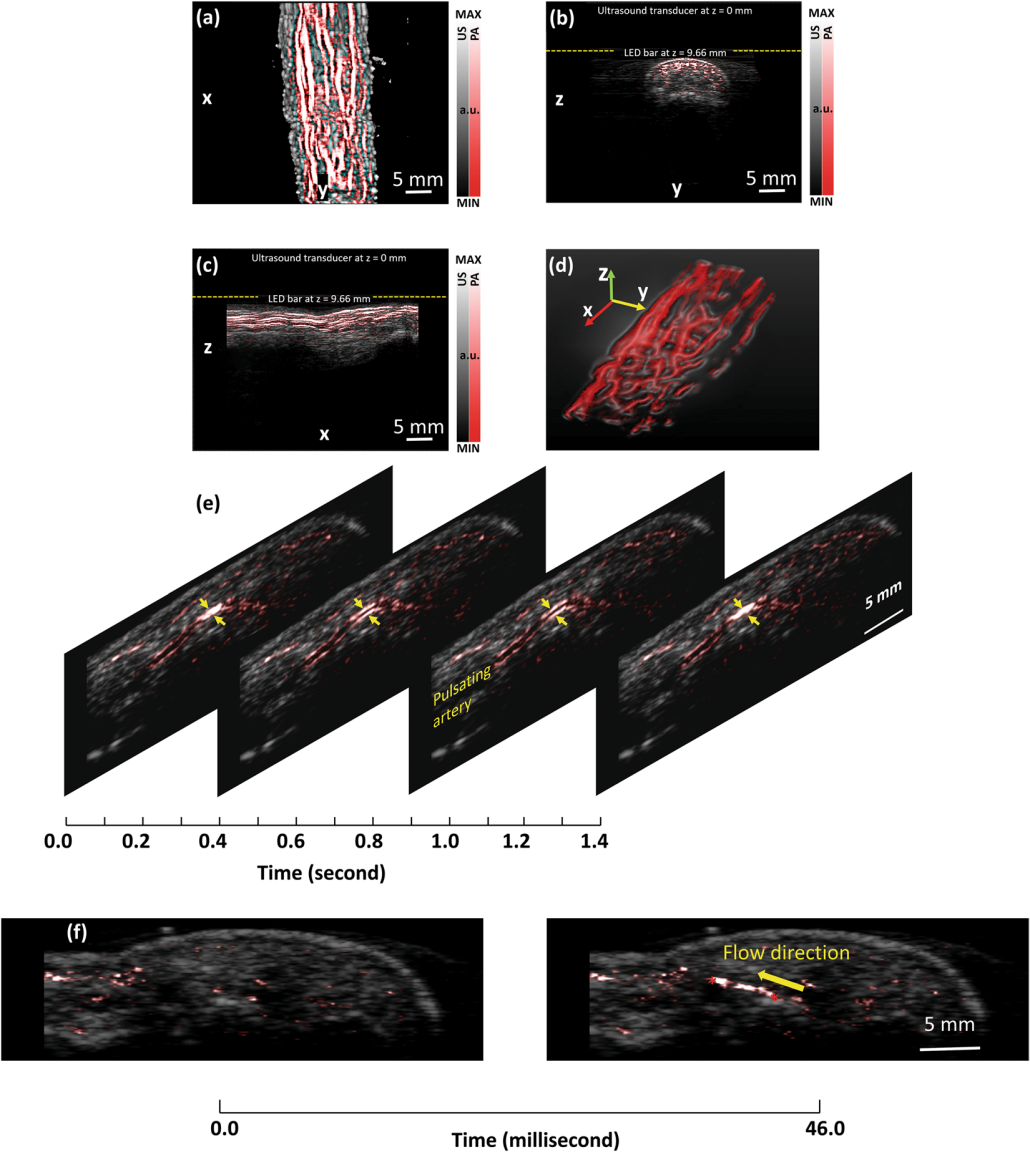
(**a**) Coronal, (**b**) axial, and (**c**) sagittal views of the 3D PA and US combined image of a human finger from a volunteer. (**d**) Perspective view of the 3D PA image showing the microvessels in the finger. 3D rendering of the vasculature is in the Supplementary Materials (Video 1). (**e**) Four frames from a video showing the cine loop of B-scan PA imaging of a human finger (index finger, sagittal section) presenting the pulsation of an artery marked by the arrows. Imaging frame rate: 10 Hz. The video is in the Supplementary Materials (Video 2). (**f**) Two frames from a video showing the cine loop of B-scan PA imaging of a human finger (index finger, sagittal section) showing the blood reperfusion into the finger after releasing of the rubber band which tied around root of the finger. (Left) The image acquired right after the rubber band releasing; (Right) The image acquired at 46 milliseconds after the rubber band releasing. The flow rate Imaging frame rate: 500 Hz. The distance between two asterisks is measured to be 4.6 mm. Blood flow direction is marked by the arrow. The video is in the Supplementary Materials (Video 3).

In this work, *via* the experiments on human finger model, we examined the performance of LED-based PA imaging for 2D and 3D mapping of subsurface microvasculature *in vivo*, including sensitivity, spatial resolution, penetration depth, and imaging speed, as well as its feasibility in sensing and quantifying the hemodynamic properties and changes such as arterial pulsation, blood reperfusion, and blood oxygen saturation. Figure 1 shows an example result of PA and US combined 3D imaging of a human finger. The 3D PA and US combined image acquired *via* the linear scan of the probe along the digit is presented along different views including coronal, axial, and sagittal. In each 2D rendering, the PA image of microvessels is presented in pseudo-color, and superimposed on the background gray-scale US image. Figure 1(d) shows perspective view of the spatially distributed vessels in the finger acquired by PA imaging. The 3D rendering of the vasculature is in the Supplementary Materials (Video 1).

Figure 1(e) shows a real-time PA and US B-scan result of a human finger along the sagittal section with a frame rate of 10 Hz. To achieve this result, two 850-nm LED bars were driven at 4 kHz repetition rate, and 384 times averaging was used. To present the arterial pulsation (as marked by the arrows), four frames from the cine loop are presented, while the video showing the entire cine loop is the Supplementary Materials (Video 2). In the pseudo-color PA images, besides the marked artery, spatially distributed microvessels in the finger up to 5-mm deep can also be recognized. Working at a higher frame rate of 500 Hz, Fig. 1(f) shows another result of real-time PA and US B-scan of a finger demonstrating the capability of this system in imaging and quantifying fast hemodynamic changes *in vivo*. Two 850-nm LED bars were driven at 16 kHz pulse repetition rate, and 32 times averaging was conducted. Both the two frames in Fig. 1(f) are from a cine loop scan showing the blood reperfusion into the finger after releasing of a rubber band which tied around the root of the finger. The left and the right images were acquired right after and at 46 milliseconds after the rubber band releasing respectively. As we can see, during a time period of 46 milliseconds (*i.e*., 23 frames), the blood signal expanded along the vessel for a total length of 4.6 mm (from right to left, as marked by the two asterisks). The speed of blood reperfusion in this vessel is quantified to be 100 mm/s. The cine loop showing the gradual process of the blood reperfusion into the finger can be found in the Supplementary Materials (Video 3, 25 times slow motion).

**Figure 2:**
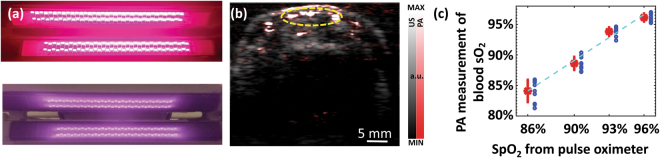
(**a**) Photo of a pair of dual-wavelength LED bars that emits 690-nm and 850-nm light alternatively. (**b**) PA (pseudo color) and US (gray scale) combined image showing the microvessels in the cross-section of a human finger. **(c)** Correlation between the dual-wavelength PA measurements of the blood sO_2_ in the finger and the SpO_2_ readouts from a pulse oximeter. A fitting line (dashed line, *y* = *a x* + *b*, *a* = 1.251, *b* = −0.234) is presented, and *R*-square of 0.9838 is achieved. At each SpO_2_ level, the asterisk on red line shows the mean, and the distance above or below it shows the standard deviation of the PA measurements.

Hypoxia is an important biomarker reflecting the onset and progression of many diseases such as cancer. It has been validated that multispectral PA imaging, by probing the spectroscopic difference between oxygenated and deoxygenated hemoglobin, can evaluate relative hemoglobin oxygen saturation and hypoxia in biological samples *in vivo*, in a non-invasive manner^16–18^. In this work, we explored the feasibility of LED-based PA imaging for measurement of blood oxygenation by using a pair of dual-wavelength LED bars which can emit 690-nm and 850-nm wavelength light alternatively, as the photo shown in Fig. 2(a). PA functional imaging of blood oxygenation in the vessels in an index finger of a volunteer was conducted. Figure 2(b) is an example PA image showing the microvessels in a cross-section of the finger, which is superimposed on the gray-scale US image. To quantitatively evaluate functional imaging result, the sO_2_ levels of the strongest 200 pixels in the region of interest (ROI) as marked by the dashed yellow circle in Fig. 2(b) are averaged. The quantified PA measurements of blood sO_2_ in the finger are then correlated with the SpO_2_ readouts from the pulse oximeter which was used as a gold standard. As shown in Fig. 2(c), the dual-wavelength PA measurements of blood oxygenation and the readouts from the pulse oximeter achieved a good correlation (*R*-square = 0.9838).

### Imaging of peripheral vasculature and response to cold exposure

Disturbance of peripheral microvascular function can be associated with many pathological conditions such as diabetes mellitus^19–24^, heart failure^25,26^, and Raynaud’s phenomenon (RP)^27,28^. RP, seen as the first manifestation in 70% of the patients with systemic sclerosis, refers to constriction of the microvessels of the hands or feet in response to cold exposure^27^. Objective evaluation of peripheral microcirculatory flow plays a key role in the characterization and treatment assessment of RP^28^. For example, it has been demonstrated that, for patients with RP, the blood flow in skin measured by laser Doppler decreases significantly after local cold exposure^29^. In this work, we explored the feasibility of LED-based PA imaging in mapping peripheral microvessels in foot, and in sensing the decrease in microvascular flow in response to cold temperature. In this study, the decrease in PA signal intensity should be caused mainly by the vasocontraction instead of the temperature change in blood which may also affect PA signal intensity via the temperature-dependent Grüneisen parameter. This is due to the fact that the temperature change in blood should be small, since the experimental duration was short and the blood in the vessels was also continuously flowing.

**Figure 3:**
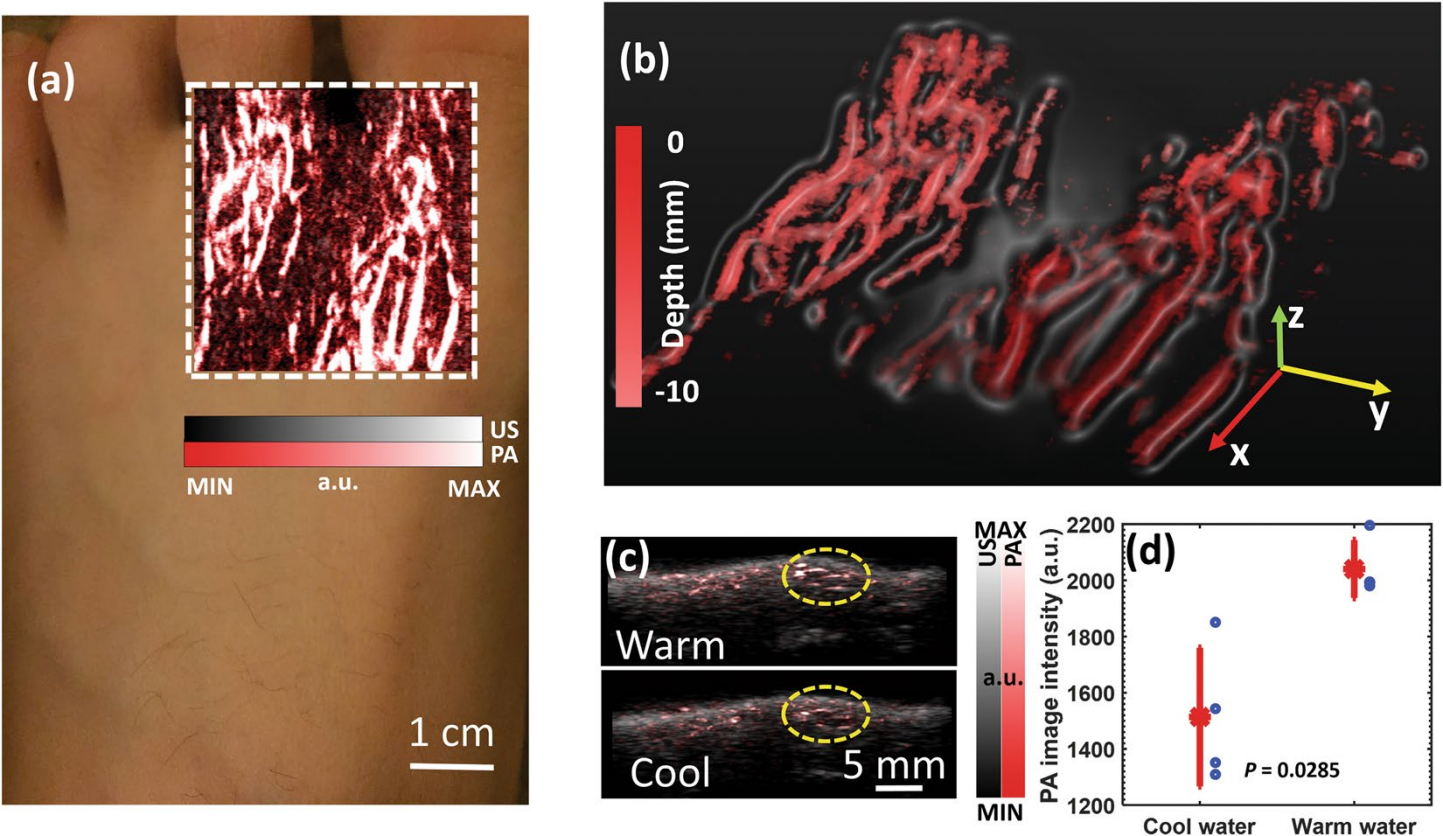
(**a**) MIP PA and US combined image showing the vasculature in the dorsal surface of a human foot. The dashed rectangle indicates the imaged area. (**b**) Perspective view image showing the 3D vasculature. The depth (*i.e*., the position along the z axis) is color encoded. (**c**) B-scan PA and US combined images of the vasculature in foot surface acquired at a temperature of 40.8 and 34.2 degree Celsius. The dashed circles indicate the regions of interest (ROI) for quantifying the change of PA image intensity in response to the local cold exposure. (**d**) Data distribution showing the averaged PA image intensities of vessels within the ROI at the two different temperatures (*i.e*., 40.8 vs. 34.2 degrees Celsius). For the PA measurements at each temperature, the asterisk on red line shows the mean, and the distance above or below it shows the standard deviation. A *p*-value of 0.0285 was achieved for a two-tailed *t*-test (n = 4), demonstrating the capability of PA imaging in detecting the change in peripheral microvascular flow in response to the local cold exposure.

PA imaging of peripheral vasculature acquired from the dorsal surface of the left foot of a volunteer is shown in Fig. 3. The dashed rectangle in Fig. 3(a) indicates the scanned area on the foot surface. With the 3D image acquired, a maximum intensity projection (MIP) PA and US combined image and a perspective view PA image with depth color-encoded are presented in Fig. 3(a,b), respectively. Spatially distributed microvessels within the depth up to 10 mm can be recognized. The 3D rendering of the acquired PA image is in the Supplementary Materials (Video 4). Figure 3(c) shows 2D B-scan images of the foot surface acquired at 40.8 and 34.2 degrees Celsius respectively. Pseudo-color PA image showing the vessels is superimposed on the gray-scale US image. The PA intensities of the blood vessels within the region of interest (ROI) marked by the dashed circle were averaged. The quantified PA measurements at the two different temperatures (40.8 degrees Celsius vs. 34.2 degrees Celsius) are compared, as shown in Fig. 3(d). With four independent measurements at each temperature (n = 4), a two-tailed *t*-test was conducted with a hypothesis that there is no difference between the PA measurements at the two temperatures. A *p*-value of 0.0285 was achieved, suggesting that the decrease in peripheral microvascular flow in response to the local cold exposure (*i.e*., temperature drop from 40.8 to 34.2 degrees Celsius) can be detected by the LED-based PA imaging.

### Imaging of inflammatory arthritis

Previous studies including those in our group have demonstrated the capability of PA technique in imaging human peripheral joints^30–34^. These relatively smaller joints are usually among the first to be affected by inflammatory arthritis. The early research findings from animal models and human subjects suggest that PA imaging holds promise for rheumatology clinic, and can provide a low-cost and non-invasive tool for early diagnosis and treatment monitoring of inflammatory arthritis^35–39^. In this research, we explored the feasibility of detecting soft tissue inflammation in human peripheral joints by using the LED-based PA system, and its capability in differentiating arthritic joints from normal joints by evaluating the enhanced microvascular flow in the synovial tissue.

**Figure 4:**
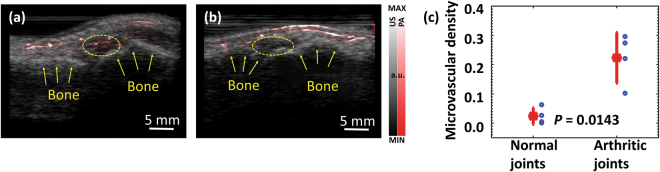
(**a**) PA and US combined image from a human MCP joint affected by inflammatory arthritis. **(b)** PA and US combined image from an MCP joint of a normal volunteer. PA images in (**a**) and (**b**) were scanned by the LED-based PA system (850-nm wavelength) using the same level of gain and the same number of signal averaging. **(c)** Quantified synovial microvascular density in the arthritic joints vs. the normal joints (n = 4 for each group). The asterisk on red line shows the mean value, and the distance above or below it shows the standard deviation. A *p*-value of 0.0143 for a two-tailed *t*-test demonstrates the capability of PA imaging in differentiating arthritic joints from normal joints based on the detecting of enhanced microvasculature as a result of inflammation.

Figure 4(a) is an example PA and US combined image from a human metacarpophalangeal (MCP) joint affected by inflammatory arthritis. The contour of the phalanges, as presented well by the US image, is marked by the arrows. In the pseudo-color PA image, besides the strong signals in the skin and the muscle which are both superficial, strong signals in the area next to the phalanges can be noticed, as marked by the dashed circle. These PA signals reflect the enhanced blood flow in the joint as the result of inflammation, which has been confirmed by the result from a Doppler US (not shown). In the PA and US combined image from a normal volunteer, as shown in Fig. 4(b), no such strong signals next to the phalanges can be noticed. Microvascular density was quantified *via* the number of PA pixels divided by the total number of pixels in the synovial area as indicated by the dashed circle. With the results from four arthritis patients and four normal volunteers, a two-tailed *t*-test was conducted with a hypothesis that the microvascular density measured by PA imaging cannot differentiate arthritic joints from normal joints. A *p*-value of 0.0143 was achieved, as shown in Fig. 4(c).

### Imaging of intraocular tumors

PA imaging has shown potential for diagnosis and characterization of cancer, especially for tumors that are relatively superficial, for example, head and neck tumors^40–45^. As a type of head and neck cancer, intraocular tumors are relatively rare but life-threatening^46,47^. Our previous study has demonstrated the capability of laser-based PA imaging in characterizing intraocular tumors by their molecular components and architectural heterogeneities^48^. In this study, we explored the feasibility in imaging intraocular tumors in intact human eye globes using LED light source.

**Figure 5:**
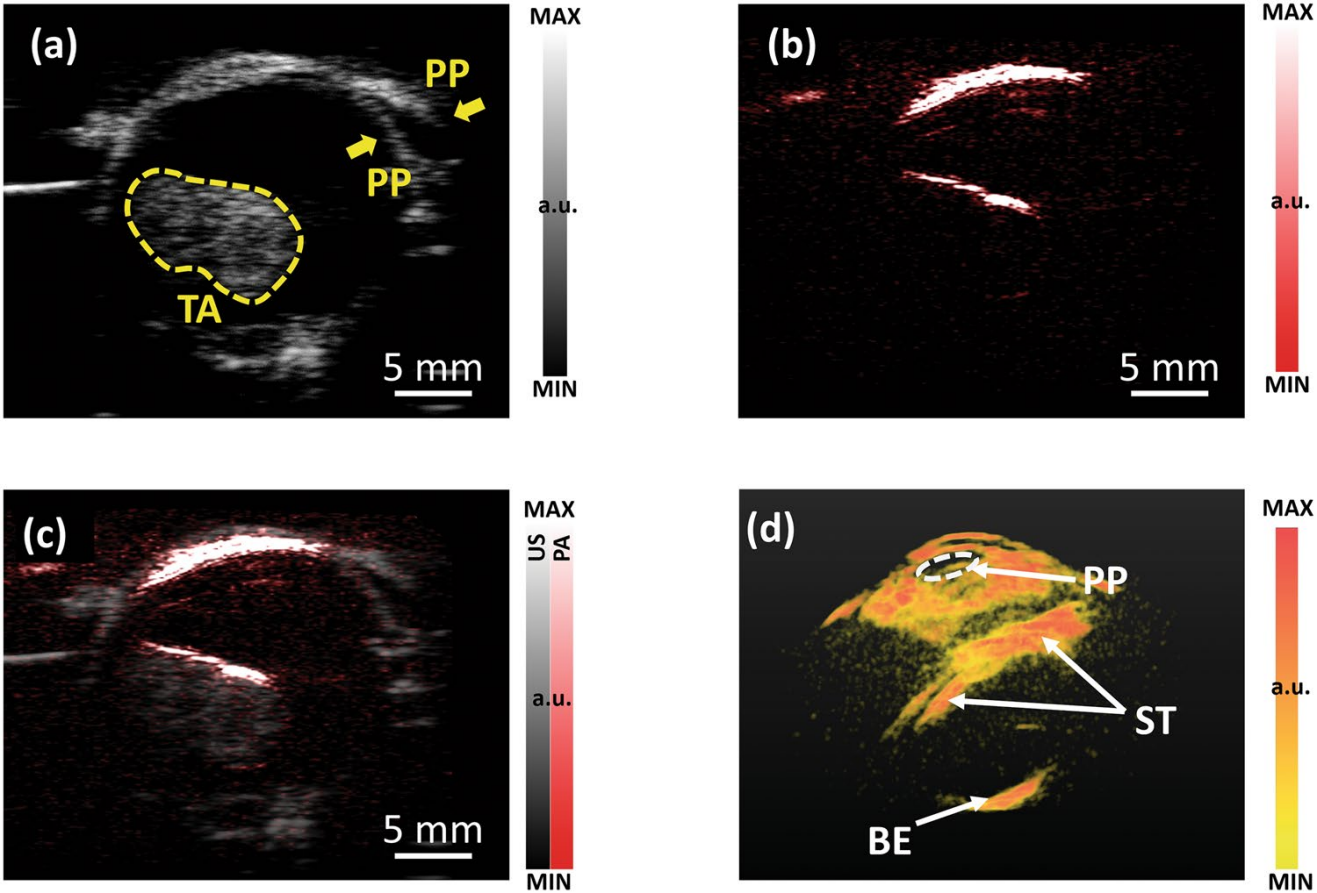
(**a**) 2D B-scan US image of an ocular globe with a choroidal melanoma tumor. The tumor area is marked. (**b**) PA image of the same imaging plane. (**c**) PA and US combined image. (**d**) Perspective view of a 3D PA image of ocular globe with a choroidal melanoma tumor. PP: pupil. TA: tumor area. ST: the surface of the tumor. BE: the back of the eye.

Figure 5(a,b) show B-scan US and PA images of an ocular globe with a choroidal melanoma tumor, respectively. Figure 5(c) shows the coregistered PA and US image. The PA result has demonstrated that LED-based PA imaging has sufficient penetration to cover the whole tumor volume. Figure 5(d) shows the perspective view of a 3D PA image of the ocular globe. In this study, LED at only one wavelength was used. LED arrays combining multiple emission wavelengths can be used in the future for observing individual molecular components. The 3D rendering of this result can be found in the Supplementary Materials (Video 5).

## Discussion

Our initial results from human subjects and organs have demonstrated that the LED PA imaging can be a potential clinical tool for the diagnosis and assessment of a variety of disease conditions associated with peripheral microvascular function, inflammatory arthritis, and head and neck cancer. The high mobility of the LED light source provides the potential PA imaging application on top of US imaging on battlefields or remote areas where resources are very limited. The user-friendly operation interface and the parallel US imaging and 3D reconstruction capability can potentially provide the clinicians with additional hemoglobin concentration and oxygenation information within the framework of conventional ultrasound diagnostic procedures. When LEDs working at other wavelengths become possible, PA imaging of other chemical information in biological samples, such as lipid or collagen content, can also be realized.

Replacing conventional class-IV lasers with LED light source may largely benefit the wide acceptance of the emerging PA imaging technology by clinic, and make it safer and more convenient for use in clinics even in resource deficient settings. Using inflammatory arthritis as an example, our previous studies employing a laser-based PA imaging system have suggested that the optical contrast presented by PA imaging is capable of detecting hyperemia and hypoxia, two important hemodynamic biomarkers of inflammation in human peripheral joints *in vivo*^39^. However, due to the high costs of class-IV lasers involved in conventional PA imaging systems, promoting this new diagnostic tool to rheumatology clinic could turn out to be challenging. The LED-based PA imaging system, with a significantly reduced cost, offers comparable image quality and sensitivity in detecting joint inflammation as those realized by laser-based PA imaging systems. The imaging depth when using the LED light source, although not as large, is sufficient for the application on rheumatoid arthritis which usually attacks the peripheral joints of human hands and feet. In addition, the smaller footprint and no need for wearing laser safety glasses during the operation of LED-based PA imaging system also make this new imaging tool more friendly to use in the rheumatology clinic rooms. Therefore, we expect that LED light source would be at least an important option in future development and clinical applications of the PA imaging technology.

The fast response and the high-speed driving of our LED arrays make the illumination pulse durations of 35–150 ns, much shorter than commonly used LED light sources and satisfying the requirement of generating PA signals efficiently. In addition, the high repetition rate of our LED arrays allows for extensive signal averaging which is crucial for boosting the SNR of the LED-based PA imaging system to a level comparable to those in the laser-based PA imaging systems without sacrificing the capability of real-time imaging. With these unique features, our LED-based PA system, when working at a pulsing repetition rate of 16 kHz, achieved a frame rate up to 500 Hz at depth up to 5 mm. This frame rate, which is an order of magnitude higher than most PA imaging systems based on solid state lasers, allows imaging and quantitative assessment of fast hemodynamic changes in biological samples, for example arterial pulsation and blood reperfusion. Although not continuously tunable, LED light source can provide many wavelength options for functional imaging, e.g., blood oxygen saturation. LED elements working at different wavelengths can be combined together and irradiate alternatively.

The LED-based PA imaging system described here is safe for both skin and eye exposure. Since LED emissions are incoherent, the ANSI safety limits for collimated laser beams do not apply. Instead, the international electrotechnical commission (IEC) 62471 is followed^49^. According to IEC 62471, the exposure limit for skin is based on thermal injury due to the temperature rise in tissue. Assuming that the illumination on the same skin area lasts continuously for 5 seconds using two 850-nm LED bars working at 4 kHz pulse repetition rate, the estimated exposure is 4.57 × 10^3^ W·m^−2^ which is below the thermal hazard limit for skin of 5.98 × 10^3^ W·m^−2^. For eye safety, two aspects need to be considered, which are retinal thermal hazard exposure limit (weak visual stimulus) and infrared radiation eye safety limit. Assuming a continuous illumination at the front of the eye for 5 second using two 850-nm LED bars working at 4 kHz pulse repetition rate, the estimated exposures are 2.92 × 10^3^ W⋅m^−2^⋅sr^−1^ for retinal thermal exposure and 4.57 × 10^3^ W⋅m^−2^ for infrared radiation exposure, both lower than the safety limits for eye of 1.34 × 10^5^ W⋅m^−2^⋅sr^−1^ and 5.38 × 10^3^ W⋅m^−2^, respectively.

Compared to powerful class-IV lasers, the energy output from the current LED arrays is relatively low. The signal averaging in the presented PA imaging partly counterbalances the reduction of illumination energy while maintaining the frame rate. Nonetheless, if the magnitudes of the acoustic signals generated by the weak illumination are significantly below the noise-equivalent pressure level of the ultrasound transducer array, averaging will not effectively remove the noises. This is the reason that penetration depth of 5 mm was found in most of our imaging results using the 10-MHz ultrasound probe. Employing ultrasound probes with higher detection sensitivity can further improve the imaging depth. As shown in Fig. S1 in Supplementary Materials, using the same LED arrays but replacing the 10-MHz probe with a 7-MHz probe, we have achieved PA imaging in biological samples with a depth up to 30 mm. Another way to further boost the SNR and the imaging depth is to perform additional signal averaging. Without sacrificing the imaging speed, the pulse repetition rate of our LED source needs to be further increased. This is technically feasible, as recently we have successfully built LED arrays with pulse repetition rate of 16 kHz, and are now working toward a higher repetition rate of 40 kHz.

Besides the emission energy level, another major difference between the pulsed LEDs and the solid state lasers is the temporal pulse width. The pulse width of our LED sources is tens of nanoseconds whereas that of the solid state lasers could be less than ten nanoseconds. The temporal pulse width imposes a limit to the spatial resolution of the imaging system. For example, the 35-ns pulse width of the 850-nm LED corresponds to a spatial resolution of 52.5 µm (=35 ns × 1500 µm/µs). One way to further increase the energy output of our LED arrays is further increasing the pulse width which can lead to degraded spatial resolution though. However, at the tomographic PA imaging scenario, the bandwidth of the transducer array is another key factor limiting the spatial resolution. For the specific case of this study, the 10 MHz transducer array corresponds to approximately 150-µm resolution. Therefore, the pulse width of the LEDs can be potentially extended to 100 ns without affecting the spatial resolution.

Despite the limitations mentioned above, this study demonstrates that short-pulse and high repetition rate LED could find application as an inexpensive and compact multi-wavelength PA excitation source for imaging superficial vascular anatomy and function.

## Materials and Methods

All the studies on human subjects have been approved by the Institutional Review Board (IRB) of the University of Michigan Medical School. Methods were carried out in accordance with the relevant guidelines and regulations. All subjects were provided written informed consent.

### LED arrays and system workflow

The LEDs (PreXion Corporation) in this study were made from various semiconductor compounds such as Aluminum Gallium Arsenide (AlGaAs), Aluminum Gallium Indium Phosphide (AlGaInP), or Indium Gallium Nitride (InGaN), all mixed together at different ratios to produce a distinct wavelength. Fig. S2(a) in Supplementary Materials shows the circuit diagram of a LED array; while the detailed layout of a LED array is shown in Fig. S2(b). To achieve an appropriate irradiating area which can be well matched with ultrasound probe, each LED bar has 4 rows of 36 LED elements (144 total elements) in order to achieve an irradiation area of 50 mm × 7 mm. A plurality of LED chips arrayed at high-density are electrically connected in series of 36 chips, and a high voltage (400 V) is applied to the LED chips to pulse-drive the LED chips, thereby flowing a large current of about 20 A to the LEDs. High-speed on/off switching by the driver is performed by a low on-resistance metal–oxide–semiconductor field-effect transistor (MOSFET). Both on-off times are 20 ns to 40 ns, and optical pulses having a width of 35 ns to 150 ns can be generated. For LED bars providing multiple wavelengths, *e.g*., 690 nm and 850 nm, as shown in Fig. 2(a), different rows working at different wavelengths can irradiate alternatively to achieve rapid multispectral PA measurements.

**Figure 6:**
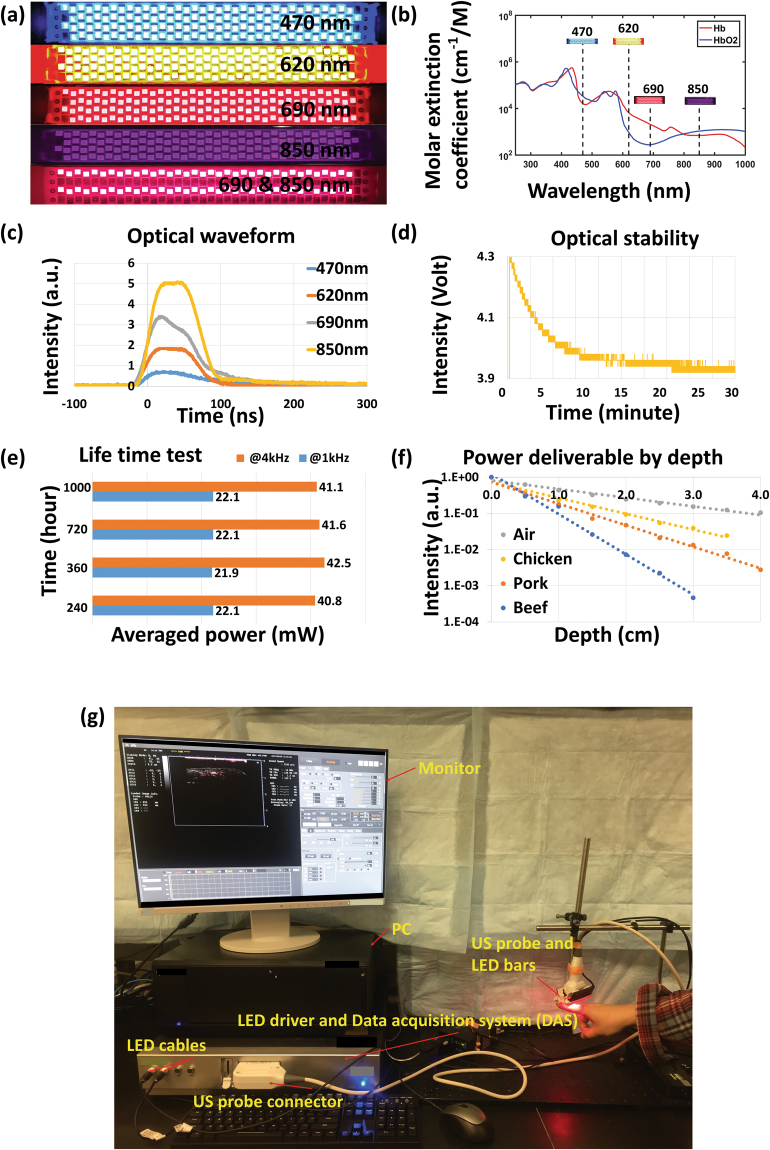
(**a**) Photograph of LED arrays working at different wavelengths (470 nm, 620 nm, 690 nm, 850 nm, and 690 nm + 850 nm dual wavelengths). As shown in (**b**), these wavelengths cover the spectral range of 400–900 nm, facilitating PA vascular imaging and quantification of hemoglobin concentration and oxygenation. The blue and the red curves show the molar extinction coefficients of the oxygenated hemoglobin (HbO2) and the deoxygenated hemoglobin (Hb), respectively. (**c**) Temporal profiles of the LED emissions at different wavelengths. (**d**) Measured output energy intensity from an LED element over a period of 30 minutes demonstrating the short-term stability. (**e**) LED lifetime test showing the average power measured at different time points over 1,000 hours, demonstrating good long-term stability. (**f**) Deliverable power emitted from a 850-nm LED array at various depths in different biological tissues, including chicken, pork, and beef. The change in deliverable power in the air as a function of depth is due to the beam divergence. (**g**) Photograph of our LED-based PA and US dual-modality imaging system.

Figure 6(a) shows the photograph of the LED arrays working at different wavelengths, including 470 nm, 620 nm, 690 nm, 850 nm, and dual-wavelength of 690 nm and 850 nm. All these wavelengths are designed within the 400–1000 nm range, as shown in Fig. 6(b), aiming at resolving the vasculatures and quantifying the hemoglobin concentration and oxygenation in biological samples. LED arrays working at other wavelengths (*e.g*., 750 nm, 810 nm, 930 nm, and 980 nm) have also been developed (not shown). The temporal profiles of the LED emissions were measured with an optical sensor (PIN photodiodes: S5973-03; diameter: 1.5 mm) placed at 5-mm distance from the LED array surface. Figure 6(c) shows the temporal response profiles of the LED arrays working at different wavelengths. The quantitative characteristics are shown in Table 1. Currently, the LED arrays working at 850 nm give the strongest energy output of 200 µJ per pulse, with a bandwidth of 35 nm. At all the wavelengths, facilitated by the fast response of the LED and the high-speed driving, LED arrays can irradiate with short pulse durations of 35–150 ns, guaranteeing the high efficiency in generating PA signals. Figure 6(d) shows the measured emission power stability of an LED working at a repetition rate of 1 kHz. The emission energy reduced by 5% after the first 10 minutes, and then maintained with an excellent stability until 30 minutes (energy fluctuation <1.2%). The long term stability and lifetime of the LED light source was also evaluated, as demonstrated in Fig. 6(e). The emission energy of LED working at both 1 kHz and 4 kHz repetition rate were stable in this test over a period of 1,000 hours. Among all the options, the LED working at 850-nm wavelength not only has the highest energy output but also enables a good imaging depth due to the excellent penetration of 850-nm light in optically scattering biological tissues. Figure 6(f) shows the deliverable power emitted from an 850 nm LED array as a function of the penetration depth in various biological tissues. The light power from the LED array was measured by an optical power meter (PD300-3W) with 5-mm^2^ receiving area. As expected, the optical power as a function of depth in each tissue follows an exponential decay as the result of the optical attenuation. In chicken breast, the measured power at 2-cm depth was about 10% of that at the surface of the LED array.

**Table 1:** Specifications of LED arrays working at different optical wavelengths.

Wavelength (nm)	850	690	620	470
Material	AlGaAs	AlGaInP	AlGaInP	InGaN
Maximal energy output (µJ)	200	80	50	60
Rising time (ns)	21	18	23	18
Falling time * except lag (ns)	36	30	39	33
Wavelength FWHM (nm)	35	21	16	20
Settable pulse width (ns)	35–150			

### PA-US dual imaging system

The PA imaging function is realized on a customized US imaging platform. Figure 6(g) shows the photograph of the LED based PA and US dual-modality imaging system. A customized US probe containing 128 elements with a pitch of 300 micrometers, an elevational dimension of 3.5 mm, and a nominal frequency of 10 MHz was used. A large element size was used in the purpose of capturing the weak PA signals. To achieve better sensitivity and imaging depth, a lower frequency 7 MHz linear array was also employed in some studies. In the US B-scan mode, the US transmission was a simple plane wave with all transducer elements. The PA signals captured were pre-amplified first by 54 dB, and then both the US and PA signals were amplified by 52 dB by customized amplifiers. Sampling rates of US and PA acquisition are 20 MHz and 40 MHz, respectively. The inclusion of an additional preamplifier for PA reception was due to the fact that PA signals are much lower in magnitude compared to US signals, and the customized low-noise pre-amplifier is the deterministic factor of the high system sensitivity.

Two LED bars were position at two sides of the US probe, with the illumination projection angle of 45° with respect to the imaging plane. Since the PA and US images were acquired by the same US transducer array, the two modalities are naturally registered. A translational stage is synchronized to the data acquisition. With accurately controlled steps, a series of 2D US and PA images reconstructed with Fourier transform analysis methods^50^ can be post-processed to reconstruct 3D images.

The data acquisition rate of PA imaging is 4 kHz for normal mode and could be up to 16 kHz for high frame rate mode by changing MOSFET working frequency. Extensive PA signal averaging was performed to achieve SNR comparable to that produced by solid state lasers. The signal averaging includes two steps, taking normal mode as an example, including 64 times by DAS and 6 times by PC, which are 384 times in total. Considering that the SNR improvement is a square root of the averaging times, 384 times averaging improves the SNR by almost 20 times. By performing extensive signal averaging, the performance of PA imaging based on two 850-nm LED bars each providing 200 µJ pulse energy is comparable to the system working with a solid state laser offering 200 µJ × 2 × 20 = 8 mJ pulse energy. The SNR in LED-based PA imaging as a function of the average number was studied, as the result shown in Fig. S3 in Supplementary Materials.

### Validation of PA imaging on phantom

**Figure 7:**
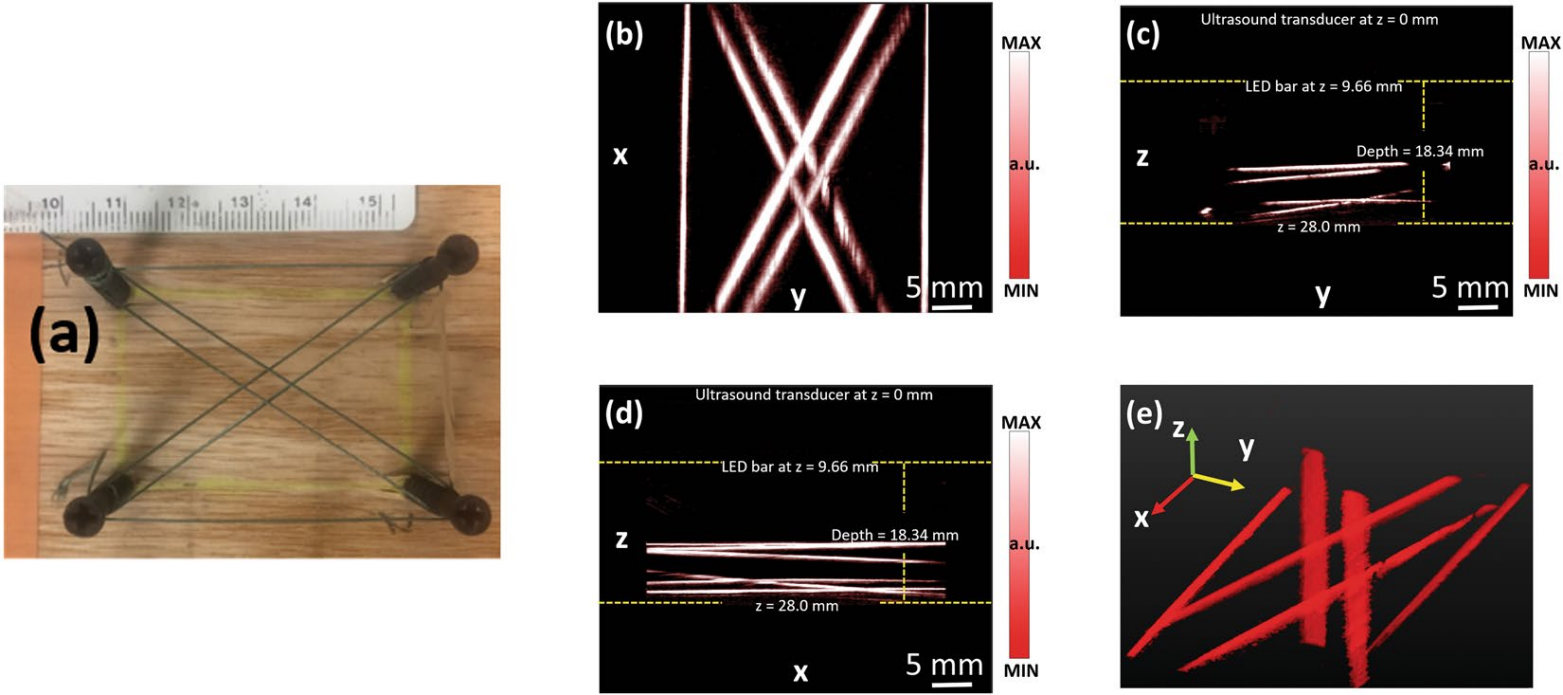
(**a**) Photograph of the phantom. (**b**) Coronal, (**c**) axial, (**d**) sagittal, and (**e**) perspective views of the acquired 3D PA image of the phantom.

To examine the performance of the PA imaging system based on the LED light source, we first imaged a phantom. The phantom shown in Fig. 7(a) was made by dark green fishing lines (diameter 0.35 mm) immersed in 1% intralipid solution for optical scattering. To acquire a 3D image of the phantom, the integrated probe with the 10-MHz linear array and two 850-nm LED bars scanned the phantom along the top surface, with a scanning step size of 0.1 mm and a total scanning range of 40 mm. The reconstructed 3D image of the phantom is presented along different views, as shown in Fig. 7(b–e). All the lines in the phantom are resolved and displayed clearly in the PA images, showing the capability of the system in resolving 3D vasculature. By studying the FWHM of the PA image intensity profile along the fishing line, after deconvolution removing the effect of fishing line size, both lateral and axial resolution of the system were measured to be approximately 200 microns. The 3D rendering of the PA image of the phantom can be found in the Supplementary Materials (Video 6).

### *In vivo* imaging of vasculature and function in human finger

For 3D imaging of vasculature in human finger, the human subject placed one hand in a water tank. With the probe oriented perpendicular to the finger, each 2D B-scan image was acquired long a cross section of the finger. For real-time 2D imaging of arterial pulsation, an index finger of a human subject was scanned along the transverse sagittal section. The position of the probe in respect to the finger was fixed during the entire measurement. The LEDs worked at 4 kHz, and PA signal averaging was 384 times. Therefore, cine loops of B-scan PA and US images were acquired at a frame rate of 10 Hz. For the result in Fig. 1(a–e), the 10-MHz linear array and two 850-nm LED bars were employed.

High speed imaging, as the result in Fig. 1(f), was performed *in vivo* on a human subject to demonstrate the capability of LED-based PA imaging in mapping and evaluating the blood reperfusion in subsurface tissues. The index finger was imaged along the transverse sagittal section, and the position of the probe in respect to the finger was fixed during the entire measurement. The LEDs worked at 16 kHz and a 500-Hz frame rate was obtained by averaging PA signals over 32 pulses. The 7-MHz linear array and two 850-nm LED bars were employed. Before imaging, the blood in the finger was squeezed out as much as possible, and then a rubber band tied the root of the finger circumferentially to temporarily hold the blood perfusion. The cine loops of PA and US images of the finger were recorded, starting before the rubber band was released and ending after the rubber band was released, with a total duration of 23.75 seconds. The finger was held still during the entire scan. In addition, working at 16 kHz pulse repetition rate, averaging over 32 pulses takes only 2 milliseconds. The motion of the finger, if any, should not cause any large artifact.

### Measurement of blood oxygenation

As shown in Fig. 6(b), the two wavelengths employed, *i.e*., 690 nm and 850 nm, are at the two sides of the isosbestic point at 797 nm, facilitating good sensitivity in multispectral PA imaging of hemoglobin oxygen saturation. Each dual-wavelength LED bar contains four rows of LED elements, with row 1 and row 3 working on 690 nm and row 2 and row 4 working on 850 nm. The LED bar emits 690-nm light and 850-nm light alternatively, forming 2 kHz repetition rate at either wavelength. Due to the fast switch between the two wavelengths (lagging time < 0.5 ms), the accuracy in quantitative imaging of blood oxygenation does not suffer from body motion. In this study, the 10-MHz linear array was employed.

This study was performed *in vivo* by imaging the vessels in the index finger of a volunteer. The finger was imaged along a transverse cross-section, and the position of the probe in respect to the finger was fixed during the entire measurement. A sensor of a pulse oximeter (V9204, Smiths Medical ASD, Inc. St. Paul, MN) clamped on the thumb of the same hand. The SpO_2_ reading from the pulse oximeter reflects the oxygen saturation in arterial blood. Assuming that the SpO_2_ and the average peripheral blood oxygenation are well correlated, the reading from pulse oximeter provides a gold standard to validate the measurement from multispectral PA imaging.

After some practice, the volunteer can manipulate the peripheral blood oxygenation level in the range of 80–100% by holding the breath. At each blood oxygenation level, the PA imaging at the two wavelengths were conducted simultaneously, both performed with a signal averaging over 1,600 pulses. Hence, each sO_2_ image was acquired within 0.8 second. With the PA images acquired at the two wavelengths, the point-by-point sO_2_ image was computed. To study the mean and the standard deviation of the sO_2_ measurements at each blood oxygenation level, 10 sO_2_ images were acquired during a time period of 0.8 × 10 = 8 seconds. During this time period, the SpO_2_ measured by the pulse oximeter varied less than 1%. As shown in Fig. 6(c), the maximum energy output from our 690-nm LEDs is only 60% of that from the 850-nm LEDs. The sO_2_ calculation method is commonly known as using the spectral unmixing approaches^16–18^. In addition, the surface reflection and the optical attenuation of the 690-nm light and the 850-nm light are different in the finger. To compensate these factors, before calculating the sO_2_ levels, a calibration of the intensities of the PA images at the two wavelengths was introduced.

### Imaging of peripheral vasculature and response to cold exposure

3D imaging of the vasculature in the dorsal surface of the foot of a volunteer was conducted by scanning the probe along the surface of the foot skin. 10-MHz linear array and two 850-nm wavelength LED bars working at 4 kHz were employed. During the entire experiment, the foot of the normal volunteer was immersed and kept still in a water tank.

For functional measurement of peripheral vasculature in response to local cold exposure, 2D B-scan images were acquired before and after the change of water temperature. The relative position of the probe in respect to the foot was kept unchanged during the study. The temperature of the water in the tank was first set at 40.8 degree Celsius. 2D imaging was repeated four times. Then, the temperature of the water was set at 34.2 degree Celsius. After about 3 minutes, 2D imaging was conducted again for four times. The imaging results acquired at two different temperatures were then compared.

### *In vivo* imaging of inflammatory arthritis

The arthritis patients involved in this study were men and women over 18 years old, with apparent swelling and pain in at least one of their finger joints. Finger joints affected by inflammatory arthritis were identified by board certified rheumatologists at the University of Michigan Rheumatology Clinic following the American College of Rheumatology (ACR) criteria. The active synovitis and enhanced blood flow in the joints were confirmed by a commercial Doppler US (z.one PRO, Zonare). To be used as control, healthy subjects with no symptoms and no history of inflammatory arthritis were also recruited. 10-MHz linear array and two 850-nm wavelength LED bars working at 4 kHz were employed.

### *Ex vivo* imaging of human eye cancer

De-identified, enucleated ocular globes containing choroidal melanoma tumors were procured at the University of Michigan Kellogg Eye Center. Malignancies were confirmed later *via* histopathology. The ocular globes, with size about 2.5 cm along both width and depth, were imaged with the LED-based PA and US system immediately after enucleation. For light illumination, the cornea area was avoided. The optical energy scattered by the sclera could provide uniform illumination at the tumor surface and assist in deep imaging. In this study, 2D and 3D PA images of ocular globe were acquired using the 10-MHz linear array and two 850-nm LED bars. The imaging probe was positioned approximately 1 cm away from the tangential plane of the eye globe surface.

### Data availability

The experimental data within this paper are available on demand by contacting the corresponding author.

## Electronic supplementary material

Supplementary Materials

video 1

video 2

video 3

video 4

video 5

video 6
