# Supplementary material for: Light Emitting Diodes based Photoacoustic Imaging and Potential Clinical Applications
Source: Sci Rep. 2018 Jun 29;8:9885. doi: 10.1038/s41598-018-28131-4 (PMC6026116; doi:10.1038/s41598-018-28131-4)
Supplement: Supplementary file 1 — Supplementary Materials [file 41598_2018_28131_MOESM1_ESM.doc]

**Supplementary Materials**

Light Emitting Diodes based Photoacoustic Imaging and Potential Clinical Applications

Yunhao Zhu^1, 2^, ^†^, Guan Xu^3^, ^†^, Jie Yuan^2, *^, Janggun Jo^1^, Girish Gandikota^3^, Hakan Demirci^4^, Toshitaka Agano^5^, Naoto Sato^5^, Yusuke Shigeta^5^, Xueding Wang^1, *^

^1^Department of Biomedical Engineering, University of Michigan, Ann Arbor, Michigan 48109, USA

^2^Department of Electronic Science and Engineering, Nanjing University, Nanjing, Jiangsu 21000, China

^3^Department of Radiology, University of Michigan, Ann Arbor, Michigan 48109, USA

^4^Kellogg Eye Center, University of Michigan, Ann Arbor, Michigan 48109, USA

^5^PreXion Corporation, Tokyo 1010041, Japan

† These authors contributed equally to this work

*Corresponding author: [yuanjie@nju.edu.cn](mailto:yuanjie@nju.edu.cn), [xdwang@umich.edu](mailto:xdwang@umich.edu)

File Name: Video 1

Description: 3D rendering of perspective view of the spatially distributed microvessels in a human finger acquired by the LED-based PA imaging.

File Name: Video 2

Description: A cine loop of 2D B-scan PA imaging of a human finger at a frame rate of 10 Hz, demonstrating the pulsation of an artery in the finger.

File Name: Video 3

Description: A cine loop of 2D B-scan PA imaging of a human finger at a frame rate of 500 Hz, demonstrating the blood reperfusion in the finger after releasing of a rubber band tied around the root of the finger. To better present the reperfusion, this video is played in a slow motion (25 times slower).

File Name: Video 4

Description: 3D rendering of PA image of the microvasculature in the dorsal surface of a human foot.

File Name: Video 5

Description: 3D rendering of PA image of a human ocular globe with a uveal melanoma tumor, demonstrating major tissue features including the pupil, the surface of the tumor and the back of the eye.

File Name: Video 6

Description: 3D rendering of the PA image acquired from a phantom.


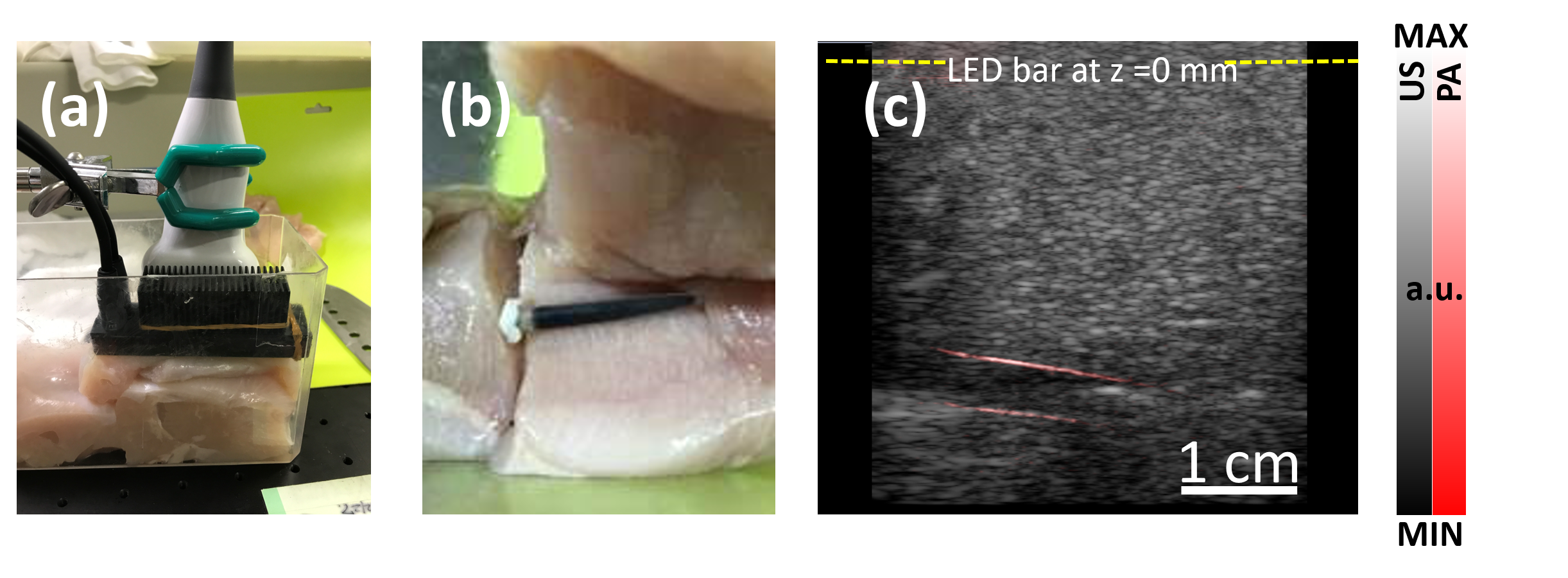


**Fig. S1**. LED-based PA imaging in deep biological tissue. **(a)** Experimental setup showing the 7-MHz liner transducer probe and the two 850-nm LED bars placed above a chicken breast tissue. **(b)** As the target object, optically transparent micro test tube (4 mm diameter, 47.5m length, and 0.5 mL volume) filled with indocyanine green (ICG) (25 mg/5 mL) was embedded at 30-mm depth in the chicken breast tissue. **(c)** 2D B-scan PA image showing the top and the bottom edges of the ICG-containing test tube, demonstrating that the imaging depth in chicken breast tissue can be up to 30 mm.


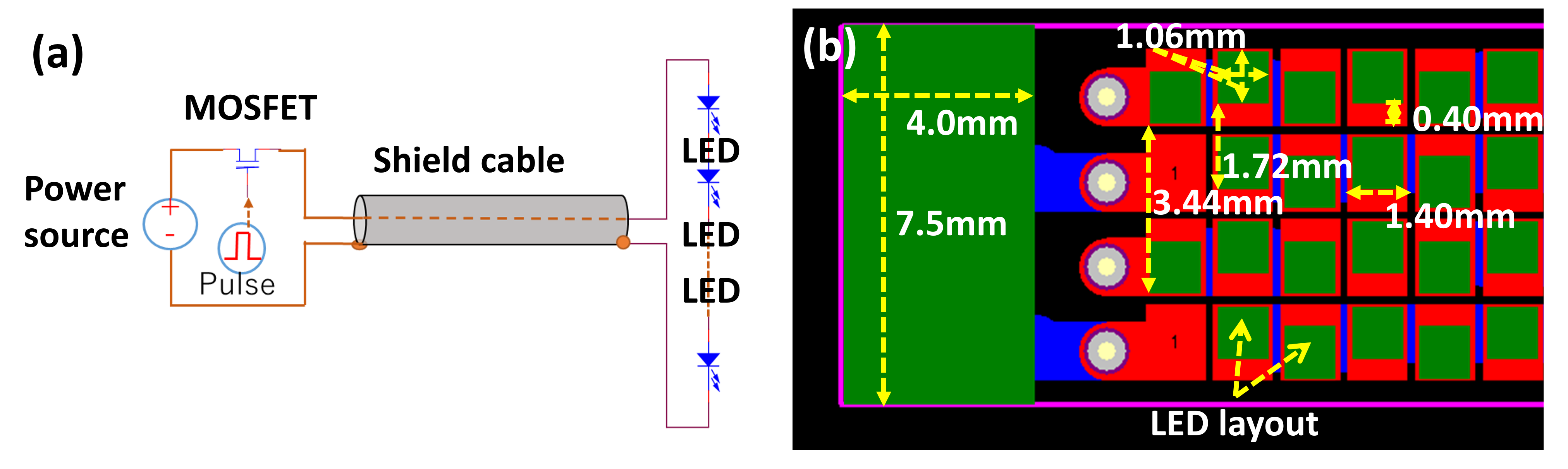


**Fig. S2**. **(a)** Schematic of LED driver. LED chips are connected in series and are switched to turn on and off at high speed by MOSFET. **(b)** Detailed layout of a LED array. Each row in the array can be switched on and off.


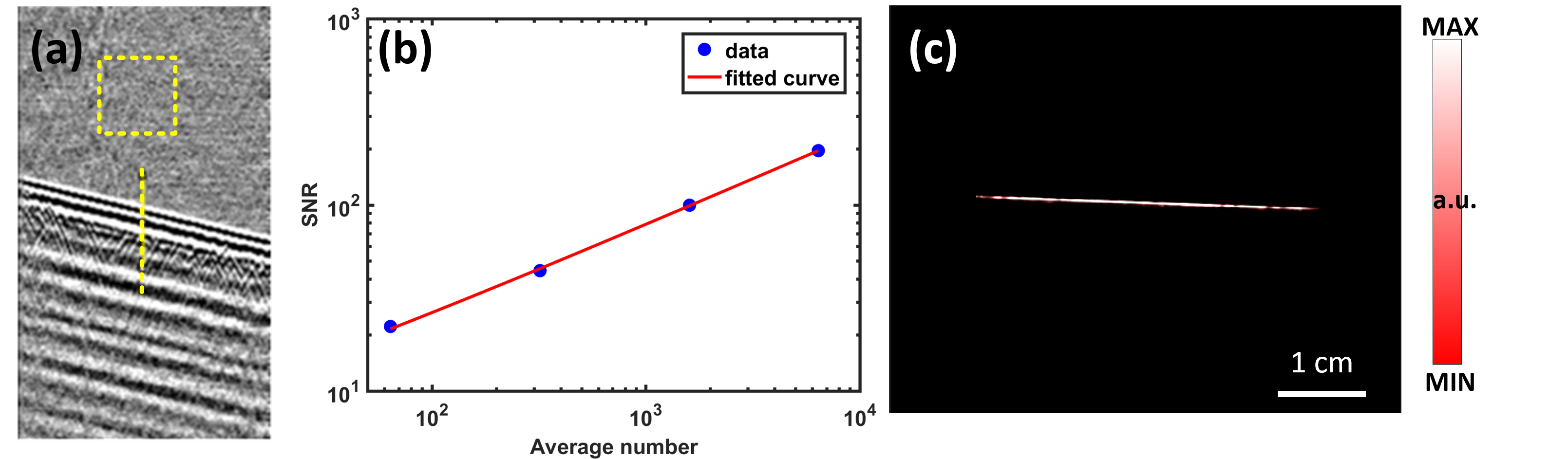


**Fig. S3**. **(a)** Radio-frequency (RF) PA signals from a steel needle received by the 128-element probe. The steel needle was placed in water, at a distance of about 2 cm from the probe surface. The average PA signal amplitude in the dashed square region gives the noise level; while the peak amplitude across the dashed line gives the signal. **(b)** PA SNR as a function of signal average number. Fitted curve:$y=a\sqrt{x}+b$. *a*=2.418, *b*=2.201, *R*-square = 0.9999. **(c)** Reconstructed PA image of the steel needle with the number of averaging of 384.
